# Myeloid-specific tristetraprolin mitigates postsurgical incisional pain by suppressing proinflammatory responses

**DOI:** 10.1007/s00011-025-02141-1

**Published:** 2025-11-26

**Authors:** Abhishek Guha, Robert E. Sorge, Ying Si, Reed Smith, Sohail M. Baig, Mohammed Amir Husain, Stacie K. Totsch, Ava M. Piper, Perry J. Blackshear, Peter H. King

**Affiliations:** 1 Department of Neurology, University of Alabama at Birmingham, Civitan 545C, 1719 6th Avenue South, Birmingham, AL 35294-0017 USA; 2 Birmingham Veterans Affairs Medical Center, Birmingham, AL USA; 3 Killion Center for Neurodegeneration and Experimental Therapeutics, University of Alabama at Birmingham, Birmingham, AL 35294 USA; 4 Department of Psychology, University of Alabama at Birmingham, Birmingham, AL USA; 5 Molecular and Cellular Biology Laboratory, National Institute of Environmental Health Sciences (NIEHS), Research Triangle Park, Durham, NC 27709 USA; 6 Departments of Medicine and Biochemistry, Duke University Medical Center, Durham, NC 27710 USA; 7 Department of Cell, Developmental, and Integrative Biology, University of Alabama at Birmingham, Birmingham, AL 35294 USA

**Keywords:** Postsurgical pain, Allodynia, Tristetraprolin, Proinflammatory mediators, RNA regulation, AU-rich element, Macrophages, Microglia

## Abstract

**Background:**

Proinflammatory mediators including COX-2, IL-1β, IL-6, and TNF-α, play major roles in the initiation of postsurgical pain. Produced primarily by activated macrophages and microglia, these mediators drive hyperexcitation of nociceptors and promote peripheral and central pain sensitization. Post-transcriptional RNA regulation is a major control point for these mediators, centering around adenine- and uridine-rich elements (ARE) in the 3’ untranslated regions of their mRNA transcripts. The ARE governs RNA stability and translational efficiency through an interaction with ARE-specific RNA binding proteins (AUBP). Tristetraprolin (TTP) is an AUBP that promotes RNA degradation and translational silencing of these mediators to suppress inflammatory responses.

**Methods:**

Mice with myeloid-specific TTP knockout or TTP knock-in underwent paw incision and were assessed for mechanical allodynia and thermal sensitivity. Molecular and cellular inflammatory responses were monitored at the site of incision, dorsal root ganglia (DRG) and lumbar spinal cord (L-SC) by qPCR, ELISA, immunohistochemistry and/or flow cytometry.

**Results:**

TTP deletion exacerbated post-incisional allodynic pain in parallel with increased edema at the site of injury and delayed wound healing but without significant effects on thermal sensitivity. There was an increase in infiltrating macrophages at the incisional site, particularly at the dermal–epidermal junction, in parallel with a robust increase in proinflammatory/pronociceptive mediators. An enhanced inflammatory response was also detected in the circulation, ipsilateral DRG and L-SC which persisted through post-incisional day 7. Conversely, TTP knock-in mice showed attenuation of allodynic pain and inflammatory responses in skin, DRG, L-SC, and circulation.

**Conclusion:**

TTP plays a critical role in mitigating postsurgical pain by tamping down peripheral, central and systemic inflammatory responses, thus identifying a new target and mechanism for future development of pain therapeutics.

**Supplementary Information:**

The online version contains supplementary material available at 10.1007/s00011-025-02141-1.

## Background

More than 80% of patients experience moderate-to-severe pain after surgical procedures which can both hamper recovery and increase the risk of opioid dependence [1, 2]. Furthermore, a small subset of patients transition to chronic pain, leading to long term disability and poor quality of life [3]. A major driver of acute postsurgical pain is the rapid surge of proinflammatory mediators, including IL-1β, IL-6, TNF-α, CCL2, COX-2 (PGE2), iNOS (NO) at the site of tissue injury, leading to direct activation and sensitization of peripheral nociceptors [4]. These mediators are also elevated in the dorsal root ganglia (DRG) and lumbar spinal cord (L-SC) on the side of injury and can promote central sensitization and chronification of pain [5, 6]. Macrophages peripherally and microglia centrally are major sources for these proinflammatory mediators and produce them in the acute phase after surgical injury [7–9]. A major node of regulation for proinflammatory mediators is post-transcriptional via adenine- and uridine-rich elements (AREs) present in the 3’ untranslated region (UTR) of the mRNA [10, 11]. The ARE regulates mRNA stability and translational efficiency through an interaction with cellular ARE binding proteins (AUBPs), ultimately regulating how much protein product is made. ARE-mediated regulation reflects a dynamic equilibrium between AUBPs that promote RNA stabilization and polysome engagement versus those that promote RNA degradation and polysome disengagement [10, 12]. The AUBP, HuR, for example, promotes expression of many proinflammatory mediators by stabilizing their mRNA transcripts and enhancing polysome engagement [10, 11]. Tristetraprolin (TTP), on the other hand, is an AUBP that suppresses expression of proinflammatory mediators and other ARE-containing transcripts by facilitating degradation of the mRNA [11, 13]. This occurs in association with polysome disengagement and loss of translational efficiency. TTP plays a key role in the resolution of inflammation and has previously been shown to ameliorate inflammation-driven diseases in animal models, including uveitis, experimental autoimmune encephalitis, arthritis and acute lung injury [14–16].

A link between pain and ARE-mediated RNA regulation was first made in the spared nerve injury model where blocking HuR either pharmacologically [17] or by gene silencing [18] mitigated post-injury pain behavior in association with blunted proinflammatory responses. Inhibition of HuR with the small molecule, SRI-42127 [19], also mitigated chronic neuropathic pain in spinal cord injury by blocking neuroinflammatory responses [20]. Here, we hypothesized that TTP modulates postsurgical pain by negatively regulating proinflammatory responses at the site of injury and more rostrally in the dorsal root ganglia and lumbar spinal cord. We tested this hypothesis with a plantar incision model of postsurgical pain in a mouse with myeloid-specific deletion of TTP [21] and one genetically programmed to upregulate TTP expression [22].

## Method

### Animal models

All animal procedures were approved by the UAB Institutional Animal Care and Use Committee (Animal Protocol Number: 20818) and in accordance with the ARRIVE guidelines. TTPΔARE knock-in (KI) and myeloid-specific TTP Knockout (KO) mice were developed by PJB as previously described [21, 22]. Briefly, TTPΔARE KI mice were generated using a targeting construct built from C57BL/6 genomic DNA, featuring a 136-bp deletion in the *Zfp36* 3′UTR (bases 1564–1699 of GenBank accession no. NM_011756) comprising an AU-rich region. Different cell types and tissues from TTPΔARE KI mice showed increased stability of TTP mRNA. On the other hand, the TTP KO is specific to myeloid cell types. This model was generated by crossing LoxP-flanked *Zfp36* mice (*Zfp36*^*fl/fl*^) with LysMcre mice, which express Cre recombinase specifically in myeloid lineage cells due to the murine M lysozyme promoter [21, 22]. Following baseline mechanical allodynia testing, 8- to 12-week-old mice were anesthetized, and a 5 mm longitudinal incision was made on the plantar surface of the left hind paw using a surgical blade. The wound was closed with two single sutures of 5–0 nylon. For the complete Freund’s adjuvant (CFA) model of inflammatory pain, a different cohort of mice received an injection of 20 µl of 100% CFA (Sigma Aldrich) into the plantar surface of the left hind paw. Mechanical testing resumed on days 1, 3, 5, 7, 9, and 11.

### Bone marrow derived macrophage, microglial and primary fibroblast cell isolation, culture and treatment

For bone marrow-derived macrophages (BMDMs), bone marrow cells were isolated from 10 week-old healthy mice. Cells were differentiated in macrophage serum-free media supplemented with 10% fetal bovine serum (FBS), 30% L929-conditioned media, and 1% penicillin–streptomycin. BMDMs were identified by flow cytometry as described [23]. Microglial cells were prepared from neonatal mice as described previously [24]. Primary fibroblasts were isolated from adult mouse and cultured based on published methods [25]. For treatment, BMDMs, microglial, and fibroblast cells were seeded in fresh media and treated with lipopolysaccharide (LPS) at a concentration of 1 μg/ml for 24 h.

### Mechanical sensitivity

Mechanical sensitivity was assessed using von Frey filaments (Stoelting) on the lateral aspect of the hind paw after injuries (at days 1, 3, 5, 7, 9, and 11). The up-down method was employed as described previously [26]. Mice were habituated to a wire mesh platform within custom Plexiglass cubicles before the assessment. In all cases, baseline mechanical sensitivity was determined prior to plantar incisional surgery. A minimum of two independent measures were recorded per hind paw.

### Radiant heat thermal sensitivity

For thermal sensitivity, mice were placed individually in cubicles atop a glass platform (IITC Inc.) and permitted to habituate for at least 60 min. A focused beam of light was aimed at the plantar surface of the hind paw, set at 20% maximum intensity. The latency to lick or shake the paw was measured to the nearest 0.1 s, with a cutoff time of 40 s.

### Wound healing and paw edema

Wound healing or closure and paw thickness were measured using digital calipers as previously described [7, 27]. Briefly, mice were anesthetized, and an incision was made on the plantar surface. Paw thickness and wound length were measured using calipers at days 0, 1, 5, and 7 after surgery. The investigator performing the analysis was blinded to the genotype of the mice.

### Flow cytometry

For flow analysis, single-cell suspensions of skin were prepared using Mouse Skeletal Muscle Dissociation Kit (Miltenyi Biotec, 130-098-305) as per manufacturer’s instruction. Immune cells were separated from myelin debris using Percoll (Cytiva, 17,089,101) gradient centrifugation as before [17]. RBCs were lysed using RBC lysis solution (Miltenyi Biotec, 130-094-183). Immune Cells were collected and incubated with a fluorescent antibody cocktail mix consisting of Zombie Aqua (fixable) viability (Invitrogen, L34957) at 1:200, CD45 PerCP/Cy5.5 (eBioscience, 45-0451-82) at 1:100, CD11b BV605 (BD Biosciences, 563,015) at 1:100, Gr1 PE/Cy7 (Biolegend, 108,416) at 1:100, Ly6C PE (Biolegend, 128,007) at 1:100, Ly6G APC (Biolegend, 127,614) at 1:100, and F4/80 APC/Cy7 (Biolegend, 123,118) at 1:100 for 30 min at 4 °C to stain myeloid cells subpopulations. After staining, cells were fixed using 2% PFA, washed and resuspended in flow sample buffer. Single cell suspensions were run on an Attune NXT Flow Cytometer (Thermo Fisher) and analyzed with FlowJo software (FlowJo v10.10.0).

### Immunoblotting, immunochemistry and ELISA

Skin and L-SC tissue samples were harvested from mice that underwent surgical incision. Tissues were lysed using T-Per kit (Thermo Fisher, 78,510), protein lysates were quantified, and immunoblotting was performed. The following antibodies were used for immunoblotting: anti-TTP (Sigma, 71,632), 1:500; anti-TTP (Cell Signaling, 71,632), 1:1500; anti-IBA1 (FUJIFILM Wako, 019-19741), 1:200; anti-COX2 (Santa Cruz Biotechnology, sc-376861), 1:1000; anti-iNOS (Cell Signaling, 13,120), 1:2000; anti-Vinculin (Abcam, ab129002), 1:3000. Vinculin was used as endogenous control which remained stable in all *in cellulo* and in vivo experiments. Immunoblots were performed with at least three replicates.

For immunohistochemistry, Fresh frozen skin and DRG sections were stained with IBA1 antibody (FUJIFILM Wako, 019-19741), 1:400, overnight at 4 °C. After washing in PBS, slides were incubated with a donkey anti-rabbit Cy3 secondary antibody (Jackson Immuno Research, 111-165-144) for 90 min at RT and subsequently counterstained with DAPI (Sigma Aldrich, 62,248) at 1:1000. Confocal imaging was done to assess macrophage infiltration. Total number of macrophages was quantified on both skin and DRG tissues. IBA1 fluorescence intensity (FI) was assessed on skin tissue. Epidermal thickness was measured as previously described [28]. Briefly, a line was drawn across the epidermis using the ImageJ line tool, and the distance between the two points that represented the top and bottom of the epidermis was then calculated using the measurement tool. Thickness of the epidermis was calculated by dividing the epidermal area by the epidermal width. To minimize sampling error, epidermal thickness was measured at several points throughout the epidermis. The investigator performing the analysis was blinded to the experimental conditions. Additional sections were stained with hematoxylin and eosin using an H&E staining kit (Abcam, ab245880) as per manufacturer’s protocol.

For ELISA, cytokines and chemokines (IL-1β, IL-6, TNFα, IL-10, CCL2 and CXCL1) were quantified in plasma samples, skin and L-SC lysates using U-PLEX mouse biomarker assays (Meso Scale Discovery, K15069M) according to the manufacturer’s instruction.

### RNA analysis

RNA was extracted either from BMDMs or from skin, dorsal root ganglia (DRG) and L-SC tissue samples using TRIzol (Thermo Fisher, 15,596,026) and/or RNASpin Mini kit (Cytiva, 25,050,072). For cell culture samples, cells were lysed using the lysis buffer provided in the RNASpin Mini kit. For skin and L-SC, tissues were lysed using T-per buffer prior to RNA isolation. For DRG samples, RNA was isolated using TRIzol method combining DRGs from at least 2–3 mice. cDNAs were synthesized using the High-Capacity cDNA Reverse Transcription Kit (Thermo Fisher, 4,368,814). RNA levels of cytokines, chemokines and other inflammatory mediators, were quantified utilizing Taqman primers (Thermo Fisher, 4,331,182) with the QuantStudio 5 Real-Time PCR system (Thermo Fisher). RPS9 (Thermo Fisher, 4,331,182) was used as internal controls.

### Statistical analysis

Statistical analyses were conducted using GraphPad Prism 10.4.0 (San Diego, CA, USA). Mechanical sensitivity scores were assessed by repeated measures analysis of variance (ANOVA) with test time (baseline, post-surgery), and sex (male, female), as variables. A multiple unpaired t-test with Welch’s correction was used for multiple comparison at each time point. For flow cytometry, qRT-PCR, and ELISA, a two-tailed unpaired *t* test was used. For IHC, a two-tailed Mann–Whitney test was used for comparison.

## Results

### Deletion of myeloid-specific TTP in mice exacerbates mechanical allodynia following paw incision

**Fig. 1:**
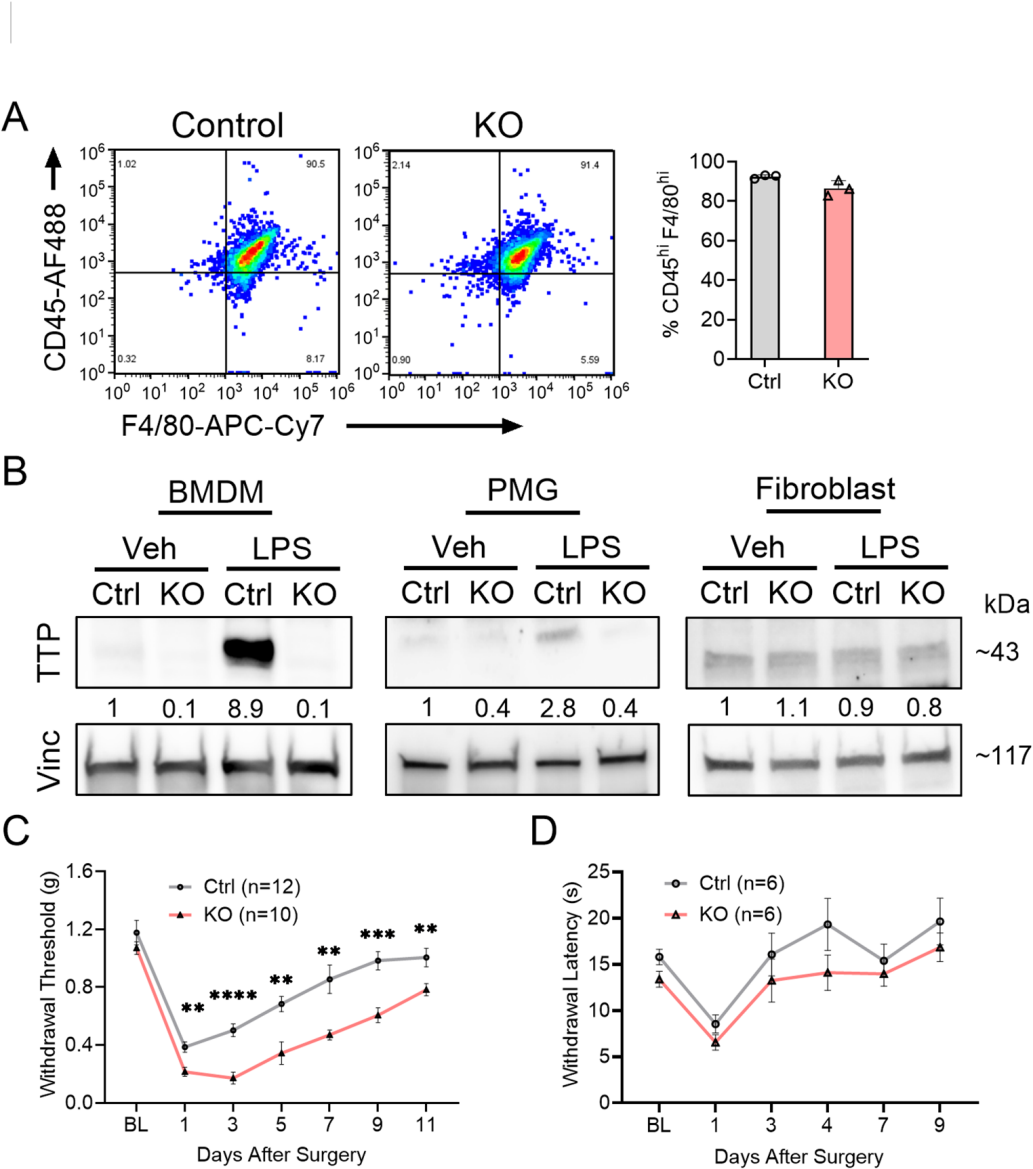
Myeloid-specific TTP deletion exacerbates mechanical allodynia in mice following paw incision. **A** Flow cytometry was conducted to identify macrophage cells derived from bone marrow of TTP KO mice. Sample gating of CD45^high^/F4/80^high^ cell populations was used to detect macrophages (left panel). Percentage of TTP KO Macrophages was compared with control (right panel), error bars represent SD. **B** Representative western blots of BMDM, primary microglia (PMG) and primary fibroblasts derived from TTP KO or floxed control mice treated with LPS. Antibodies used are shown to the left of the blot. Densitometry ratios of band intensities (TTP/Vinculin normalized to vehicle control) are shown below the TTP blots. **C** TTP KO and control mice underwent plantar paw incision and were tested for mechanical allodynia (withdrawal thresholds) at 1-, 3-, 5-, 7-, 9- and 11 days post-surgery. **P < 0.01, ***P < 0.001, ****P < 0.0001, Multiple comparison unpaired t-test with Welch’s correction. Data represent mean ± SEM. A repeated-measures ANOVA revealed significant time effects (F (6,120) = 52.63, *p* < 0.0001) and genotypic effects (F (1,20) = 57.36, *p* < 0.0001). **D** TTP KO and Control mice were tested for thermal sensitivity (withdrawal latency) at 1-, 3-, 4-, 7-, and 9 days post-surgery

We first assessed TTP expression in BMDM (identified by flow cytometry, Fig. 1A), primary microglia (PMG), and adult fibroblasts from control and TTP KO mice after LPS treatment. Western blot analysis showed near absence of TTP expression in BMDM and PMG from TTP KO mice, whereas expression in adult fibroblasts remained unaltered (Fig. 1B). LPS treatment increased TTP expression in PMG and BMDM but not fibroblasts. There was a concomitant induction of key inflammatory mediators—IL-1β, IL-6, iNOS, and CXCL1—in control cells following LPS treatment, which was significantly enhanced in TTP KO BMDM (Supplementary Fig. 1A). To investigate the role of TTP in regulating post-surgical incisional pain, we assessed mechanical allodynia in TTP KO mice at multiple time points. There was enhancement of mechanical allodynia in TTP KO mice (*p* < 0.0001; Fig. 1C), with no sex dimorphism (Supplementary Fig. 1B). This was in conjunction with increased edema at the site of injury and delayed wound healing (Supplementary Fig. 11). However, no significant difference in thermal sensitivity was observed between controls and TTP KO mice (Fig. 1D). We also tested TTP KO mice in a Complete Freund’s Adjuvant (CFA)-induced inflammatory pain model and observed enhanced allodynia (Supplementary Fig. 1C). Taken together, these findings indicate that TTP deletion in myeloid cells exacerbates mechanical hypersensitivity after surgical injury in both male and female mice which persists at 11 days post incision.

### TTP deficiency leads to increased macrophage infiltration at the site of paw incision

**Fig. 2:**
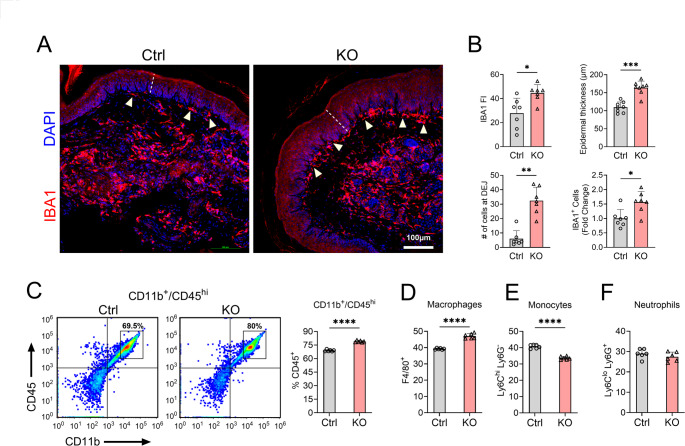
TTP deletion increases macrophage infiltration at the site of paw incision. **A** Ipsilateral skin tissue sections were immunostained with an anti-IBA1 antibody and counterstained with DAPI. White arrows indicate the IBA1^+^ macrophages present at dermal–epidermal junction (DEJ). Scale bar, 100 µm. **B** Fluorescence Intensity (FI) of IBA1 immunoreactivity (left upper panel), epidermal thickness (right upper panel), number of IBA1 at DEJ (left lower panel) and total number of IBA1^+^ cells (right lower panel) were quantified in 7 controls and 7 TTP KO samples. *P < 0.05, **P < 0.01, ***P < 0.001; two-tailed Mann–Whitney test, error bars represent SD. **C** Flow cytometry of incisional site was performed to identify myeloid cells in TTP KO mice. Sample Gating of CD11b^+^/CD45^high^ was used for the detection of myeloid cells other than microglia (left). Percentage of CD11b^+^/CD45^high^ myeloid cells in TTP KO mice was quantified and compared with control (right panel). **D–F** CD11b^+^/CD45^high^ myeloid cells were further sorted into subpopulations, such as Macrophages (Gr-1^low^/F4/80^hi^), Monocytes (Gr-1^mi^/Ly6C^high^/Ly6G^−^), and Neutrophils (Gr-1^high^/Ly6C^low^/Ly6G^+^). Data points in the graph represent individual mouse samples. ****P < 0.0001; Unpaired two-tailed t-test, error bars represent SD

Immunohistochemical analysis revealed a 1.6-fold increase in IBA1 + cells at the injury site (Fig. 2A, B; *p* = 0.03), with a 1.4-fold increase in total IBA1 fluorescence intensity (Fig. 2A, B;* p* = 0.017). Interestingly, TTP KO mice showed an eightfold increase in IBA1 + cells at the dermal–epidermal junction (Fig. 2A, B). In parallel with the increased inflammatory infiltrate in TTP KO mice, there was a 1.5-fold increase in epidermal layer thickening compared to floxed control littermates 24 h after surgery (Fig. 2A, B and Supplementary Fig. 2). Sham TTP KO mice showed no pathological changes compared to sham control mice (Supplementary Fig. 3). Further analysis of myeloid populations using flow cytometry revealed increases in CD45highCD11b + cells (~ 14.5%) and macrophage infiltration (~ 20%) at the surgical site in TTP KO mice (Fig. 2C, D,* p* < 0.0001). Additionally, TTP KO mice exhibited a ~ 21% reduction in monocytes, indicating active differentiation into skin-infiltrating macrophages (Fig. 2E and Supplementary Fig. 4; *p* < 0.0001). There was no difference in neutrophils (Fig. 2F and Supplementary Fig. 4). Taken together, these findings demonstrate a more pronounced inflammatory infiltrate at the site of incision in TTP KO mice.

### Myeloid deletion of TTP leads to increased production of inflammatory mediators at the paw incision site

**Fig. 3:**
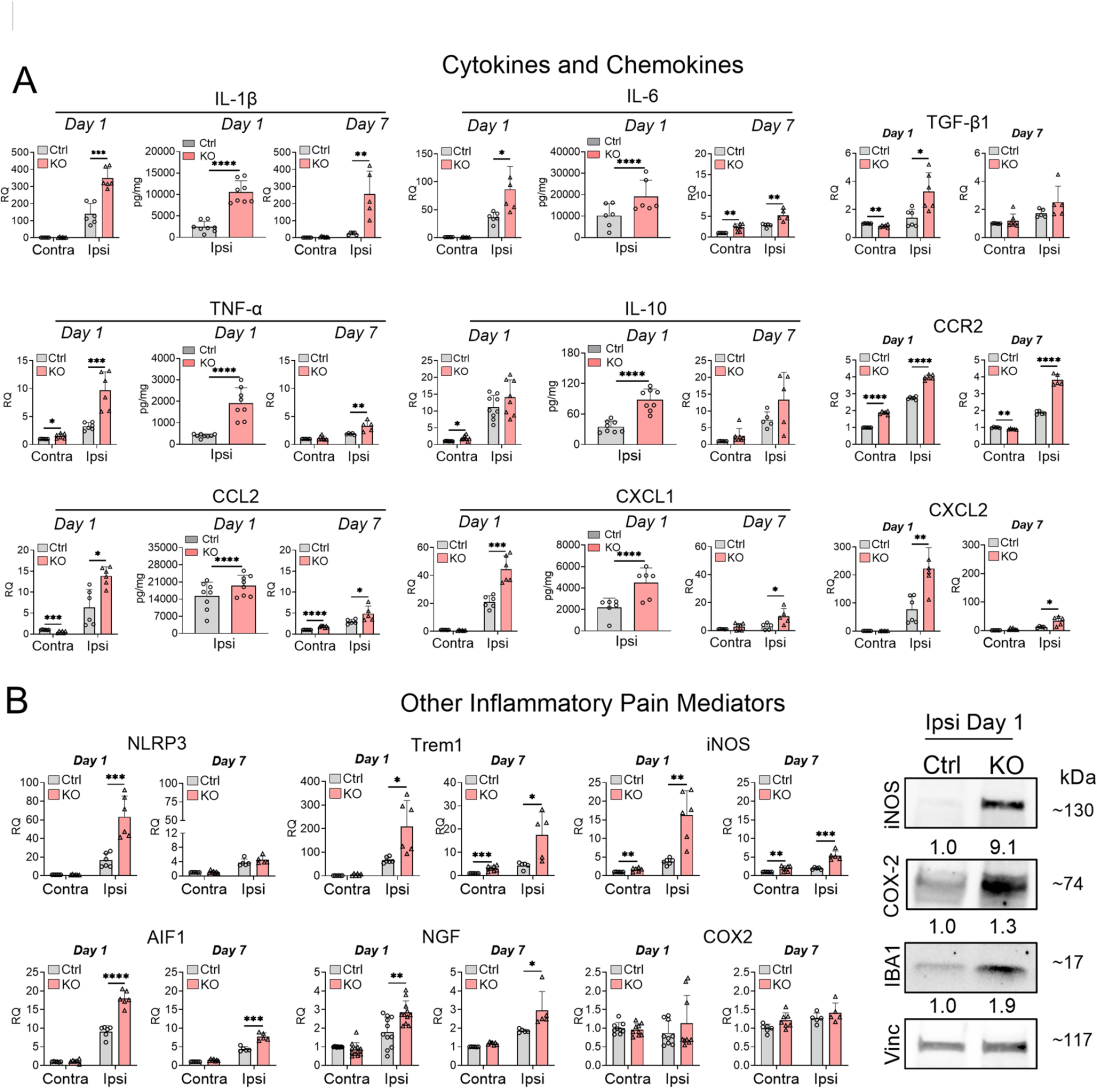
TTP deletion increases expression of inflammatory mediators at the site of paw incision. **A** Mice underwent surgical incision under anesthesia and were euthanized at day 1 and day 7 post incision. Skin tissues contralateral and ipsilateral to the site of incision were harvested and analyzed qRT-PCR and ELISA for expression of cytokines and chemokines. *P < 0.05, **P < 0.01, ***P < 0.001, ****P < 0.0001; unpaired two-tailed t-test, error bars represent SD. **B** mRNA and protein levels of other inflammatory mediators involved in pain modulation were measured by qRT-PCR and western blotting. Antibodies are shown to the left of the blots. Densitometry ratios of band intensities (Target protein/Vinculin normalized to vehicle control) are shown below each blot. *P < 0.05, **P < 0.01, ***P < 0.001, ****P < 0.0001; unpaired two-tailed t-test, error bars represent SD

To elucidate potential mechanisms underlying enhanced skin inflammation and macrophage infiltration in TTP KO mice, we characterized the expression of 15 key inflammatory mediators implicated in pain modulation, including 9 cytokines and chemokines (Fig. 3). In control mice, at the site of surgical injury (ipsilateral side), there was a significant but variable induction of most inflammatory mediators 1 day after incision. The induction was strongest with IL-1β, IL-6, IL-10, CXCL1, CXCL2, NLRP3, Trem1, and AIF1 while TNF-α, CCR2, CCL2, iNOS, and NGF showed more modest induction (Fig. 3A, B). TGF-β1, COX2, and iNOS essentially showed no induction. With the exception of IL-10 and COX2, TTP KO mice showed further enrichment of inflammatory mediators on the ipsilateral side at day 1, including IL-1β (2.5-fold,), IL-6 (2.4-fold), TNF-α (3-fold), TGF-β1 (2.3-fold), CCR2 (1.4-fold), CCL2 (2.2-fold), CXCL1 (2.1-fold), CXCL2 (2.9-fold), NLRP3 (3.8-fold), iNOS (4.3-fold), Trem1 (3.1-fold), AIF1 (2-fold), and NGF (1.6-fold) (Fig. 3A, B). Although there was no change in TTP expression in the skin of control mice 1 day after surgical incision, TTP was significantly reduced in both ipsilateral and contralateral sites in TTP KO mice compared to control (Supplementary Fig. 10). By day 7, the overall expression of these mediators decreased in both control and KO mice, except for IL-1β which was persistently increased in KO mice (twofold compared to the contralateral side) with no reduction compared to post-incision day 1 (Fig. 3A, B). COX2 mRNA levels remained unchanged at day 7. Increases in protein levels at day 1, as measured by ELISA or western blot, were in line with RNA changes for iNOS (~ 9.1-fold), TNF-α (~ 4.8-fold, *p* < 0.0001), IL-1β (~ 4.2-fold, *p* < 0.0001), IL-10 (~ 2.5-fold, *p* < 0.0001), CXCL1 (~ 2-fold, *p* = 0.006), IL-6 (~ 1.9-fold, *p* = 0.04), and IBA1 (~ 1.9-fold) in TTP KO mice on the ipsilateral site. For COX2, RNA levels did not change on the ipsilateral side, but the protein levels increased by 1.3-fold (Fig. 3B) which further enhanced (~ 3.9-fold) by day 7 post-incision (Supplementary Fig. 5). In summary, TTP depletion in myeloid cells leads to a robust induction of proinflammatory mediators at the surgical incision site at day 1, with two key pain-driving mediators (IL1β and COX2) persisting through 7 days.

### Myeloid deletion of TTP augments macrophage infiltration and selective upregulation of inflammatory mediators in DRG and L-SC following paw incision

**Fig. 4:**
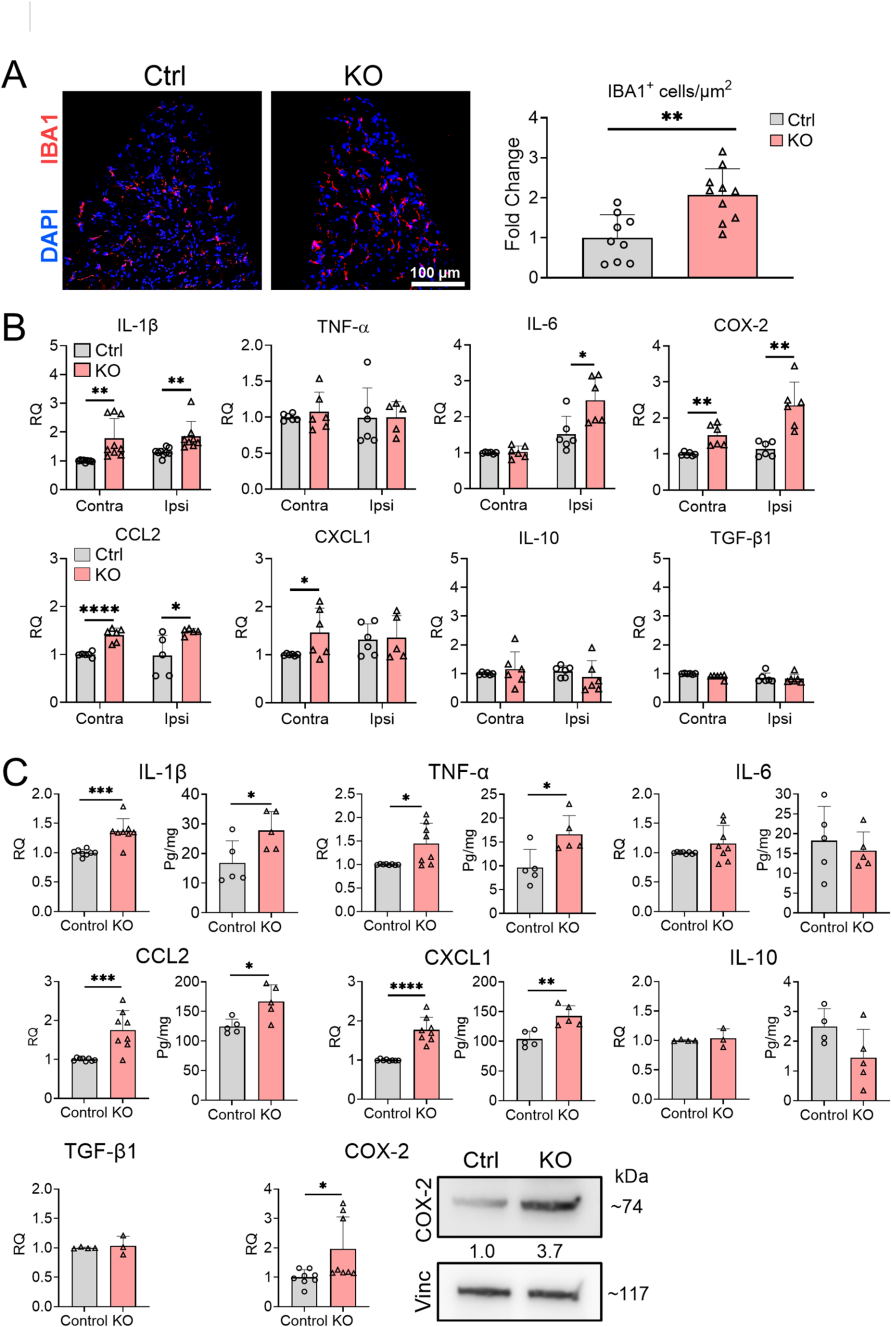
TTP deletion triggers inflammatory responses in the DRG and L-SC following paw incision. **A** Ipsilateral DRG tissue sections were immunostained with an anti-IBA1 antibody and counterstained with DAPI. Scale bars, 100 µm. The number of IBA1^+^ cells/area in TTP KO DRG were quantified and plotted as fold-change compared to control littermates (set at 1). Data points represent individual mouse samples. **P < 0.01; two-tailed Mann–Whitney test, error bars represent SD. **B** mRNA levels of selected cytokines and chemokines were measured in DRG samples, both contralateral and ipsilateral to the incision site, at 1 day post-incision. Each data point represents pooled DRGs from 2–3 mice. *P < 0.05, **P < 0.01, ****P < 0.0001 unpaired two-tailed t-test, error bars represent SD. **C** mRNA and protein levels of the same targets were measured in L-SC samples using qRT-PCR, western blotting and ELISA. Data points represent individual mouse samples. *P < 0.05, **P < 0.01, ***P < 0.001 and ****P < 0.0001; unpaired two-tailed t-test, error bars represent SD. Densitometry ratios of band intensities (COX-2/Vinculin normalized to vehicle control) are shown below the COX-2 blot

Given that paw incisional injury can induce inflammatory responses in DRG and L-SC that modulate pain sensitization [9], we investigated the impact of TTP deletion in those neural tissues. Immunohistochemical analysis of DRG ipsilateral to the incisional site revealed a significant increase in macrophage infiltration in TTP KO mice compared to littermate controls (Fig. 4A), with a 2.1-fold increase in IBA1 + cells (Fig. 4A, *p* = 0.0014). We quantified expression of 8 inflammatory mediators, including IL-1β, IL-6, TNF-α, IL-10, TGF-β1, CCL2, CXCL1, and COX2. At day 1 post-incision, comparing ipsilateral to contralateral DRG, there was minimal to no induction of these mediators in littermate controls (Fig. 4B). In TTP KO mice, there was minimal enrichment in the ipsilateral DRG which included IL-1β (1.4-fold, *p* = 0.0067), IL-6 (1.6-fold, *p* = 0.017), CCL2 (1.5-fold, *p* = 0.03), and COX2 (2.1-fold, *p* = 0.0014). Neither IL-10 nor TGF-β were induced. TTP expression remained significantly reduced similar to the incision site (Supplementary Fig. 10). At day 7, there continued to be minimal enrichment of several inflammatory mediators which now included IL-10 and TGF-β1 (Supplementary Fig. 6). IL-1β and IL-6, however, reverted back to control levels. We next assessed L-SC tissues. We found a significant ~ 20% increase in TTP expression in the L-SC of control mice at day 1 post surgical incision, whereas TTP was significantly reduced (~ 13%) in TTP KO mice compared to control (Supplementary Fig. 10). There was significant enrichment (*p* < 0.05) of mRNA and protein expression (respectively) for IL-1β at 1.4-fold and 1.7-fold, TNF-α at 1.4-fold and 1.7-fold, CCL2 at 1.8-fold and 1.3-fold, CXCL1 at 1.8-fold and 1.4-fold, and COX2 at 2-fold and 3.7-fold (Fig. 4C). IL-6 and TGF-β1, were not enriched at the RNA level. Neither IL-10 mRNA nor protein levels were altered. At day 7, mRNA enrichment persisted in TTP KO mice for IL-1β (2.2-fold), TNF-α (1.3-fold), CXCL1 (3.4-fold), and COX-2 (1.2-fold) (Supplementary Fig. 7). Taken together, myeloid-specific TTP deletion leads to selective and persistent enrichment of inflammatory responses in the DRG and L-SC after surgical incision but to a lesser degree than the incision site.

### Paw incision enhances systemic inflammation in TTP KO mice

**Fig. 5:**
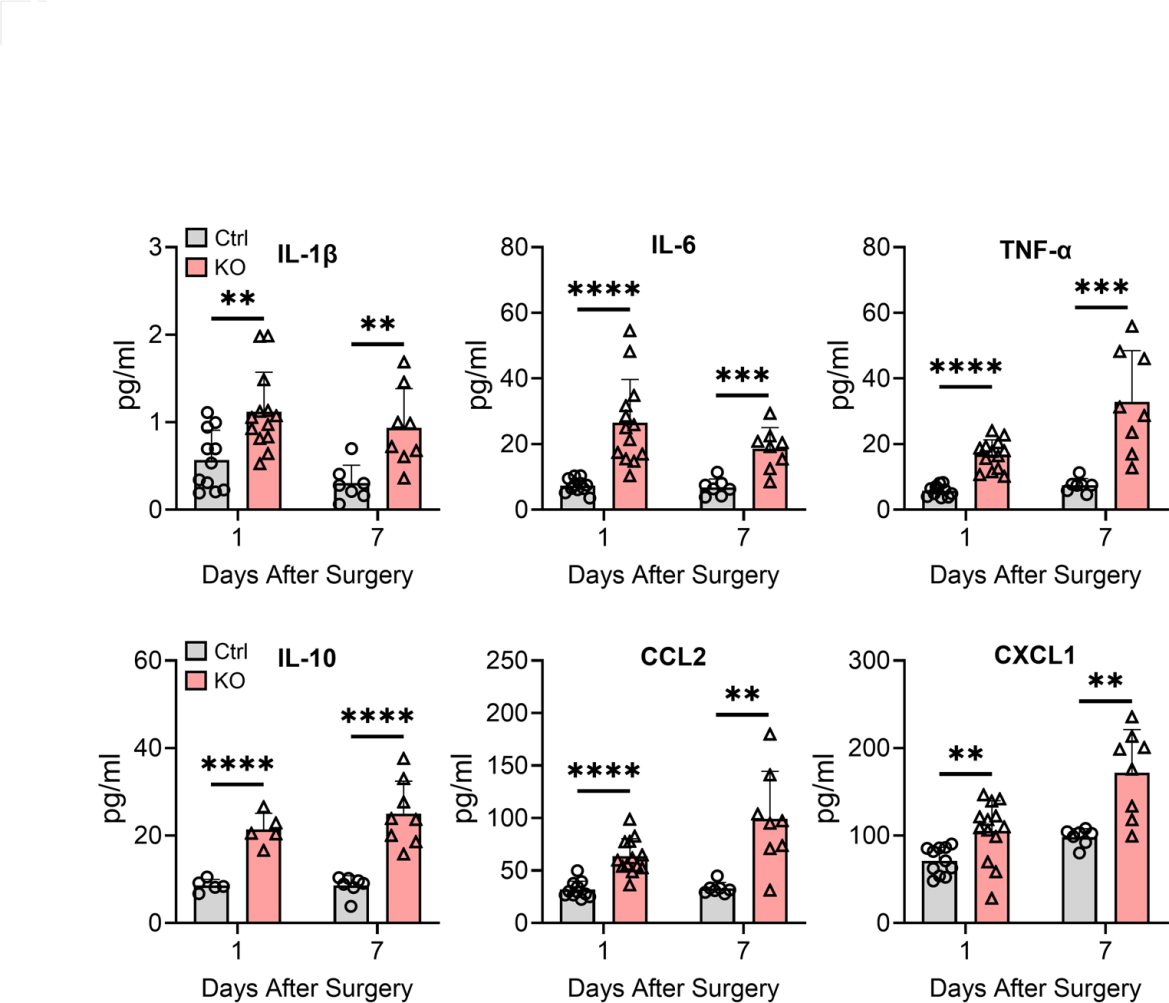
Circulating inflammatory mediators are increased in TTP KO mice after surgical incision. Plasma samples were collected from control as well as TTP KO mice 1- and 7 days post incision. ELISA was used to quantify cytokines and chemokines in plasma samples obtained. Data points represent individual mouse samples and error bars represent SD. **P < 0.01, ***P < 0.001, and ****P < 0.0001; unpaired two-tailed t-test

As circulating proinflammatory mediators, including IL-1β and IL-6, can further sensitize nociceptors peripherally and centrally [29, 30], we assessed plasma samples by ELISA at 1 and 7 days after paw incision (Fig. 5). TTP KO mice had a significant increase (*p* < 0.05) in plasma levels at day 1 and day 7 (respectively) for IL-1β at 2-fold and 3.1-fold, IL-6 at 3.6-fold and 2.7-fold, TNF-α at 2.9-fold and 4.4-fold, CCL2 at 2-fold and 3-fold, CXCL1 at 1.5-fold and IL-10 at 2.5-fold and 2.9-fold. Collectively, these results suggest that myeloid deletion of TTP leads to an intensified and persistent systemic inflammatory response.

### Overexpression of TTP mitigates post-incisional mechanical allodynia and inflammatory responses

**Fig. 6:**
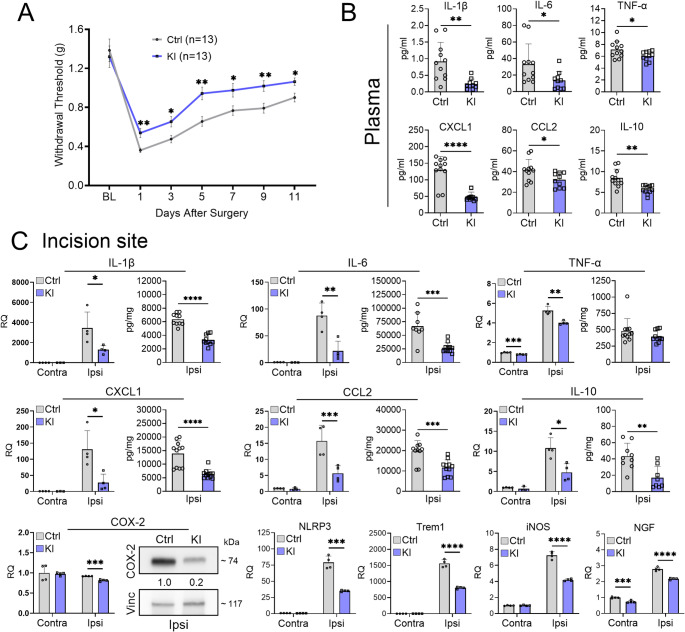
TTP overexpression attenuates post-incisional mechanical allodynia and reduces proinflammatory responses.** A** TTP ΔARE KI and flox control littermates underwent plantar incision and were tested for mechanical allodynia (withdrawal thresholds) at 1, 3, 5-, 7-, 9-, and 11 days post-surgery. *P < 0.05, and **P < 0.01, multiple comparison unpaired t-test with Welch’s correction. Data represent mean ± SEM. A repeated measure ANOVA revealed significant time effects (F (6,144) = 48.35, *p* < 0.0001) and genotypic effects (F (1,24) = 15.84, p = 0.0006) **B** Plasma samples were collected from control as well as TTP KI mice 1 day post incision and analyzed by ELISA. Data points are individual mouse samples and error bars represent SD. *P < 0.05 **P < 0.01, and ****P < 0.0001; unpaired two-tailed t-test.** C** Skin tissues contralateral and ipsilateral to the incision were harvested at 1 day. RNA and protein levels of cytokines and chemokines were analyzed by qRT-PCR, ELISA and western blotting. *P < 0.05, **P < 0.01, ***P < 0.001, ****P < 0.0001; unpaired two-tailed t-test, error bars represent SD. Densitometry ratios of band intensities (COX-2/Vinculin normalized to vehicle control) are shown below the COX-2 blot

We tested a TTP knock-in (KI) mouse model in which TTP is expressed at significantly higher levels due to genetic deletion of a destabilizing ARE in its 3’ UTR (Supplementary Fig. 8A) [22]. Following paw incision, TTP KI mice showed attenuation of mechanical allodynia compared to littermate controls that persisted through day 11 (Fig. 6A). A separate analysis of male and female mice revealed no sex dimorphism (Supplementary Fig. 8B). Next, we assessed plasma samples and found that KI mice had suppressed levels of IL-1β (74%), IL-6 (60%), IL-10 (33%), TNF-α (15%), CCL2 (23%), and CXCL1 (62%) relative to controls (Fig. 6B). At 1 day post incision, we assessed expression of key inflammatory mediators at the incisional site. In TTP KI mice there was suppression of multiple inflammatory mediator mRNAs, including IL-1β (63%), IL-6 (75%), TNF-α (24%), IL-10 (56%), CCL2 (64%), CXCL1 (79%), NLRP3 (55%), iNOS (42%), Trem1 (48%), NGF (22%1), and COX2 (12%) compared to littermate controls (Fig. 6C). Protein analysis from the same skin samples showed a concomitant downregulation of IL-1β (48%), IL-6 (60%), IL-10 (60%), CCL2 (40%), and CXCL1 (54%) in KI mice (Fig. 6C). COX-2 was suppressed by 5-fold as determined by western blot (Fig. 6C).

In ipsilateral DRG, there was suppression of IL-1β (65%1), IL-6 (17%,), CCL2 (13%, *p* = 0.022) and AIF1 (6%) (Supplementary Fig. 9A). No changes were observed with iNOS, COX2, IL-10 or TGF-β1. Interestingly, there was some suppression of IL-1β, IL-6, CCL2, AIF1, and TGF-1, albeit modest, in the contralateral DRG. Protein analysis from L-SC of TTP KI mice demonstrated a significant reduction in IL-1β (64%), TNF-α (60%2), CCL2 (21%7), and CXCL1 (41%) (Supplementary Fig. 9B). Together, our findings demonstrate that upregulation of TTP significantly attenuates mechanical allodynia following surgical incision in parallel with suppression of key inflammatory mediators in skin, DRG, lumbar spinal cord, and plasma.

## Discussion

Treatment options for postsurgical pain remain limited and are fraught with the risks of side effects that hamper recovery and the potential for opioid dependence [9]. With over 200 million surgical procedures performed world-wide, there is a strong need for identifying new mechanisms of postsurgical pain that could be therapeutically targeted [31]. We have identified TTP, an AUBP that dampens myeloid inflammatory responses, as a modulator of postsurgical pain. Our findings lay the groundwork for future development of therapies that can target TTP and this level of gene regulation to improve clinical outcomes.

A link between ARE-mediated RNA regulation and pain was first made in the spared nerve injury (SNI) model of chronic neuropathic pain by our group and the Galeotti group [17, 18]. Those studies targeted HuR, an AUBP that promotes proinflammatory responses by increasing mRNA stabilization and translational efficiency [10, 11]. With either antisense-mediated suppression of HuR [18] or direct pharmacological inhibition with SRI-42127 [17, 19], these studies reported sustained reduction in post-SNI mechanical allodynia. This phenotype was associated with suppression of post-injury inflammatory mediators in the lumbar spinal cord, many of which overlap with those found to be regulated by TTP, including IL-6, iNOS, COX-2, IL-1β, CCL2 and TNF-α. More recently, SRI-42127 treatment after thoracic spinal cord injury blocked neuropathic pain in parallel with a robust suppression of central and peripheral proinflammatory responses [20]. The overlap of these proinflammatory targets with those that drive other pain syndromes, including chemotherapy, infection (e.g., Herpes Zoster) and diabetic neuropathy, underscores the importance of ARE-mediated regulation as a potential target for therapeutic development [32].

ARE-mediated RNA regulation is crucial for the rapid control of inflammatory responses, either facilitating the production of inflammatory mediators through stabilization of their mRNA transcripts and promoting polysomal engagement (translational efficiency) or suppressing them by RNA degradation and polysome disengagement [11, 12, 16]. The net effect is governed by a balance between negatively regulating AUBPs such as TTP and KSRP and positively regulating AUBPs like HuR. AUBPs compete for ARE binding, and the equilibrium can be shifted when one or more loses or gains access to the ARE. With TTP deleted in macrophages, microglia and other myeloid cells, the balance is shifted toward a net-positive regulation of proinflammatory mediators. Since macrophages and monocytes are major drivers of the acute inflammatory response at surgical incision sites [33], the resultant effect is a marked enhancement of an inflammatory milieu which promotes nociceptor activation [4, 34]. Neutrophils also likely had reduced TTP expression (and a potentially increased proinflammatory response) due to the high efficiency of LysM-Cre recombinase in this population [35], but prior immune cell depletion studies suggests that this population of myeloid cells does not contribute to the development of mechanical hypersensitivity after incision [34]. Consistent with this observation, TTP KO mice in our investigation did not show a change in neutrophil infiltration. We postulate that the early infiltration of TTP-deleted macrophage/monocyte cells triggered an amplified inflammatory response, including the recruitment and activation of additional macrophages, leading to higher levels of pronociceptive factors. This amplification is further accentuated by pattern-recognition receptors NLRP3 and Trem-1 which increased by 3 to 4-fold in TTP KO mice (Fig. 3) [36, 37]. These genes are strongly regulated by ARE-mediated pathways: negatively by TTP (and KSRP) [38, 39] and positively by HuR [38, 40, 41].

TTP-regulated factors such as IL-1β, TNF-α, IL-6, and COX-2 are “master” mediators of inflammation [42–44], and can induce other cells to produce proinflammatory and pronociceptive factors in the milieu of surgical injury including keratinocytes, axons, Schwann cells (e.g., NGF), and dendritic cells [8, 45–50]. The 8-fold increase in “lining” macrophages at the dermal-epidermal border underscores this possibility due to the proximity of basal keratinocytes. Interestingly, deletion of TTP in keratinocytes leads to spontaneous development of local and systemic inflammatory responses due mainly to over production of TNF-α [51]. In our investigation, there was a robust increase in circulating proinflammatory mediators, including IL-1β and IL-6, which can further sensitize nociceptors peripherally and centrally [29, 30]. Because of amplified responses, the pattern of upregulated pronociceptive mediators observed at the site of incision in TTP KO mice extends beyond the direct effect of deleted TTP. NGF, for example, is upregulated after incisional injury and promotes peripheral sensitization [47, 48, 52]. It was significantly higher with TTP deletion but has not been reported to be regulated by TTP. The molecular profile in TTP KO mice also included a significant increase in the anti-inflammatory mediator, IL-10, at site of incision and L-SC and conversely a decrease in TTP KI mice. This pattern is consistent with its recognized role as a negative regulator of IL-10 via AREs in the 3 ‘UTR [53, 54]. IL-10 has been shown in a number of pain models (injury and inflammatory-based) to be antinociceptive mainly by suppressing the production of pronociceptive cytokines such as TNF-α [55, 56]. A major mechanism for this suppression, however, is at the post-transcriptional level which requires TTP [54, 55]. Thus, IL-10 would have less anti-inflammatory effects in TTP-depleted myeloid cells (as supported by the robust increase in proinflammatory cytokines). Taken together, the perturbed inflammatory milieu after incisional injury reflects both direct and indirect effects of TTP deletion in myeloid cells.

**Fig. 7:**
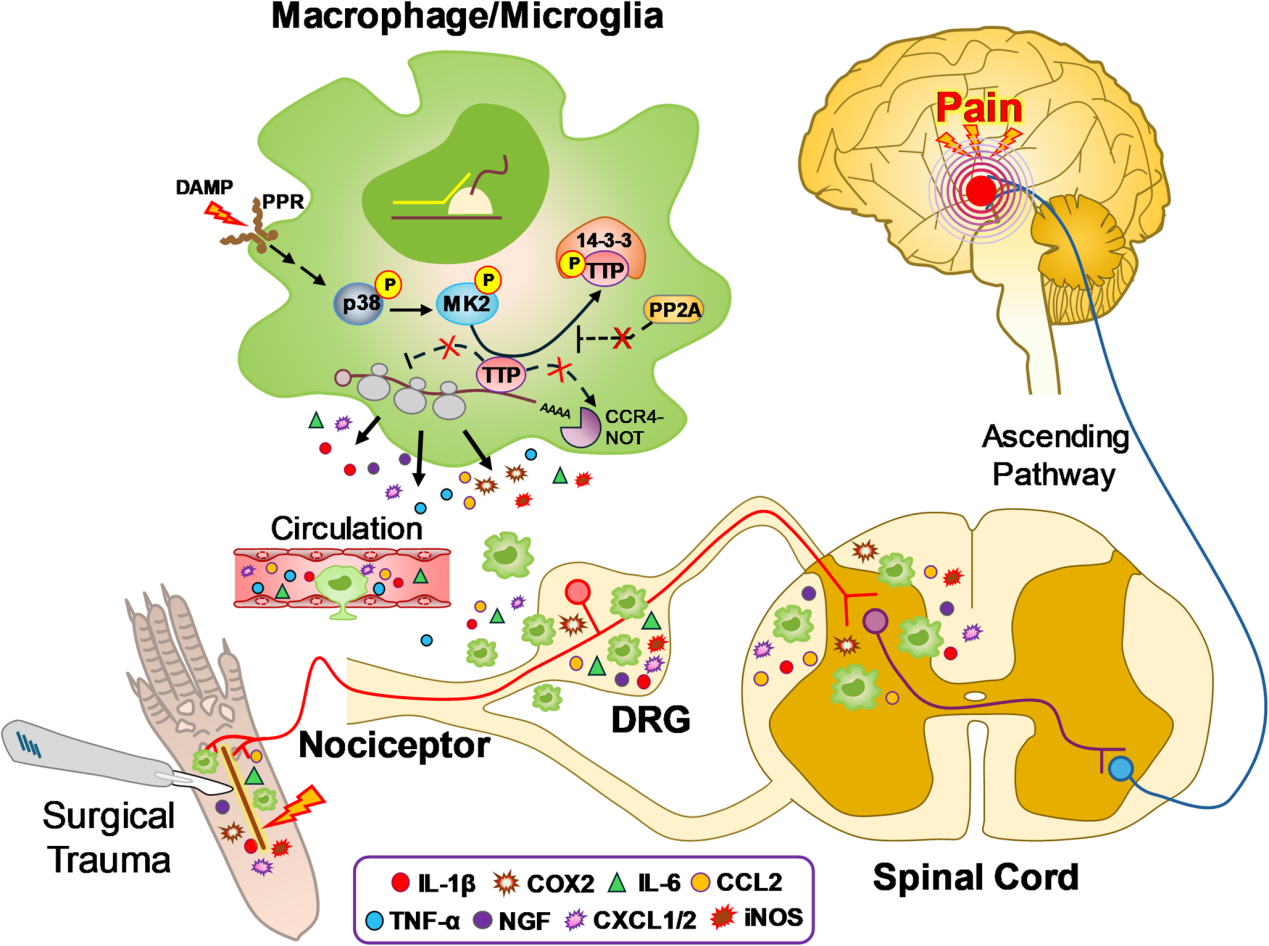
Proposed model showing the role of myeloid-specific TTP in post-surgical incisional pain. Under basal conditions, TTP suppresses production of inflammatory mediators in macrophages/microglia by binding to AU-rich elements (AREs) within the 3′UTR of target mRNAs, promoting RNA degradation via CCR4–NOT complexes and dissociation from polysomes. Following surgical incision, p38 MAPK is activated through pattern recognition receptor (PRR) signaling (e.g., Toll-like receptor engagement with DAMPs). This leads to MK2-mediated phosphorylation of TTP, causing its dissociation from proinflammatory mRNA AREs and sequestration by 14-3-3 proteins, while simultaneously inhibiting PP2A-dependent TTP dephosphorylation [70]. These events stabilize proinflammatory mRNAs and enhance polysome association, resulting in robust production of proinflammatory and pronociceptive mediators. Taken together, TTP inactivation is the key molecular switch that drives activation of macrophages and microglia in the periphery, dorsal root ganglia (DRG), and spinal cord, leading to increased mediator release that promotes hyperexcitation of pain-transmitting sensory neurons and central sensitization

On the other hand, in the TTP KI mouse, removal of its destabilizing ARE leads to enhanced expression, thus favoring its access to proinflammatory mRNAs [22]. This resulted in suppression of the very same proinflammatory mediators at the site of surgical incision. This deletion has been shown to suppress the expression of proinflammatory mediators in other disease models including dermatitis, arthritis and autoimmune encephalitis resulting in mitigation of tissue injury [15, 16]. Phosphorylation of TTP (or KSRP) by p38/MK2 signaling also pushes the equilibrium toward positive ARE-mediated regulation by promoting TTP complex formation with 14-3-3 and sequestering it away from the mRNA (Fig. 7) [57, 58]. p38 signaling is activated after plantar incision and plays a crucial role in the initiation of nociceptor sensitization and mechanical allodynia, and an inactivation of TTP may contribute to this process (Fig. 7) [59, 60]. Further support of the shifting ARE-binding dynamic is derived from experiments targeting HuR [10, 11, 61]. Blocking HuR nucleocytoplasmic translocation and/or its RNA binding prevents access to the mRNA in the cytoplasm resulting in enhanced TTP (and/or KSRP) binding and subsequent suppression of proinflammatory mediators [10, 61]. In previous reports from our group, for example, the small molecule HuR inhibitor, SRI-42127, was shown to block its cytoplasmic translocation in activated microglial cells and astrocytes leading to a suppression of proinflammatory mediators and pain responses in SNI and spinal cord injury [17, 20, 24]. Of note, COX-2 mRNA expression at the incision site changed minimally or not at all in TTP KO or KI mice, but protein levels increased significantly with TTP deletion and decreased with TTP upregulation. This RNA/protein dissociation likely reflects modulation of translational efficiency, a level of ARE-mediated regulation distinct from RNA degradation [11].

In the paw incision model, there is typically resolution of mechanical hypersensitivity 7–12 days after incision in parallel with the resolution of inflammatory responses including infiltration of immune cells and cytokine levels [34, 62, 63]. We observed ongoing enhanced mechanical allodynia at 11 days in TTP KO mice suggesting a possible impairment of pain resolution. Increased edema and delayed wound healing occurred in TTP KO mice in the same time frame and may have contributed to this persistent allodynia (Supplementary Fig. 11). We observed persistent elevation (7 days post incision) of key pronociceptive cytokines, chemokines and other mediators in the incision site, circulation and/or lumbar spinal cord including IL-1β, IL-6, TNF-α, iNOS, NGF, CCL2, and CXCL1. This molecular pattern highlights the important role TTP plays in the resolution of inflammation [16], which is underscored by the suppressed levels of proinflammatory factors observed in KI mice. It is unclear whether TTP KO mice will experience chronic allodynia (and chronic inflammation) beyond 11 days, but prior studies identified persistent activation of p38 in the spinal cord (and not peripherally) as a key component for the transition to chronic pain [62]. Loss of TTP functionality in the L-SC due to persistent p38-driven phosphorylation may contribute to this transition. Interestingly, TTP deletion did not exacerbate thermal hyperalgesia after incisional injury reinforcing prior reports that suggest these pain modalities have different underlying molecular mechanisms [64–69]. It is unclear, however, what target(s) in the different nociceptive pathways may be modulated by the altered secretome of TTP-depleted myeloid cells either centrally or peripherally.

## Conclusion

TTP plays a direct role in mitigating postsurgical pain responses through its suppressive effect on proinflammatory responses at the site of incision, circulation and neural tissues. Enhancing TTP function, either by increasing its expression or blocking its phosphorylation, represents a rational approach to the development of new pain therapies after surgery.

## Supplementary Information

Below is the link to the electronic supplementary material.

Supplementary Material 1

## Data Availability

The authors confirm that the data supporting the findings of this study are available within the article and its supplementary materials. Raw data are available from the corresponding authors upon request.
