# Supplementary material for: Myeloid-specific tristetraprolin mitigates postsurgical incisional pain by suppressing proinflammatory responses
Source: Inflamm Res. 2025 Nov 26;74(1):172. doi: 10.1007/s00011-025-02141-1 (PMC12657561; doi:10.1007/s00011-025-02141-1)
Supplement: Supplementary file 1 — Supplementary Material 1 [file 11_2025_2141_MOESM1_ESM.pdf]

Supplementary Figure 1

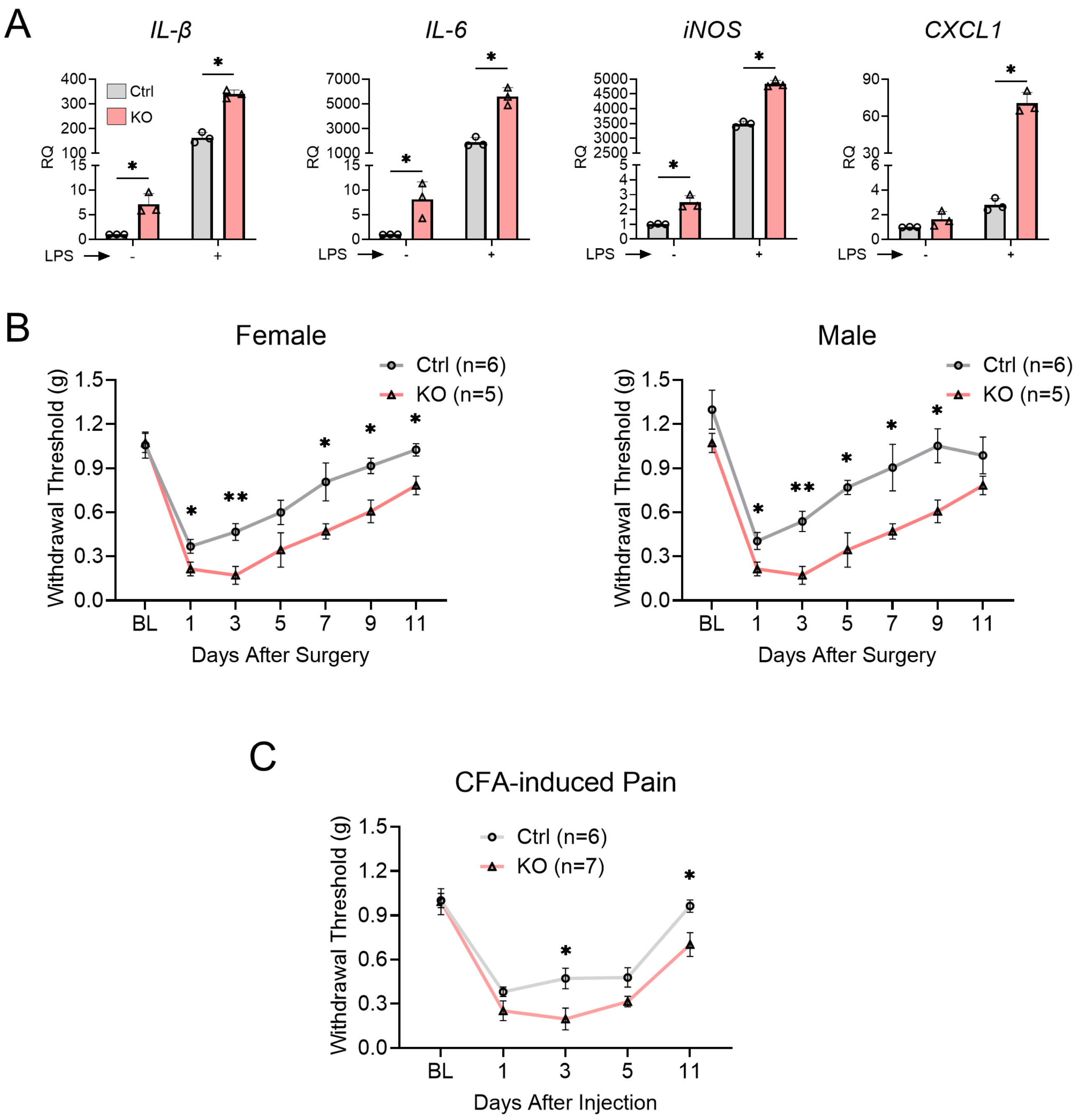

Supplementary Figure 2

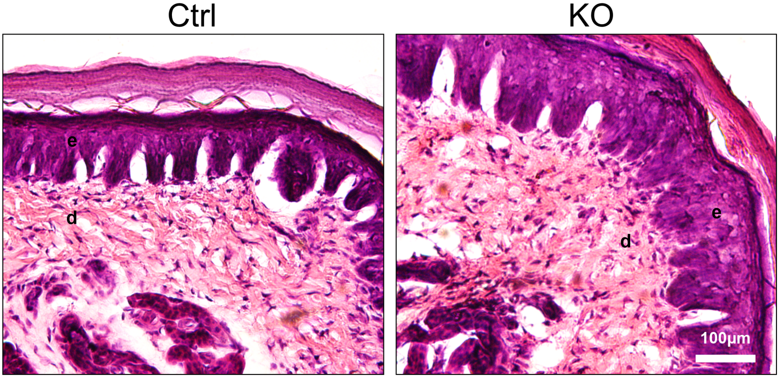

Supplementary Figure 3

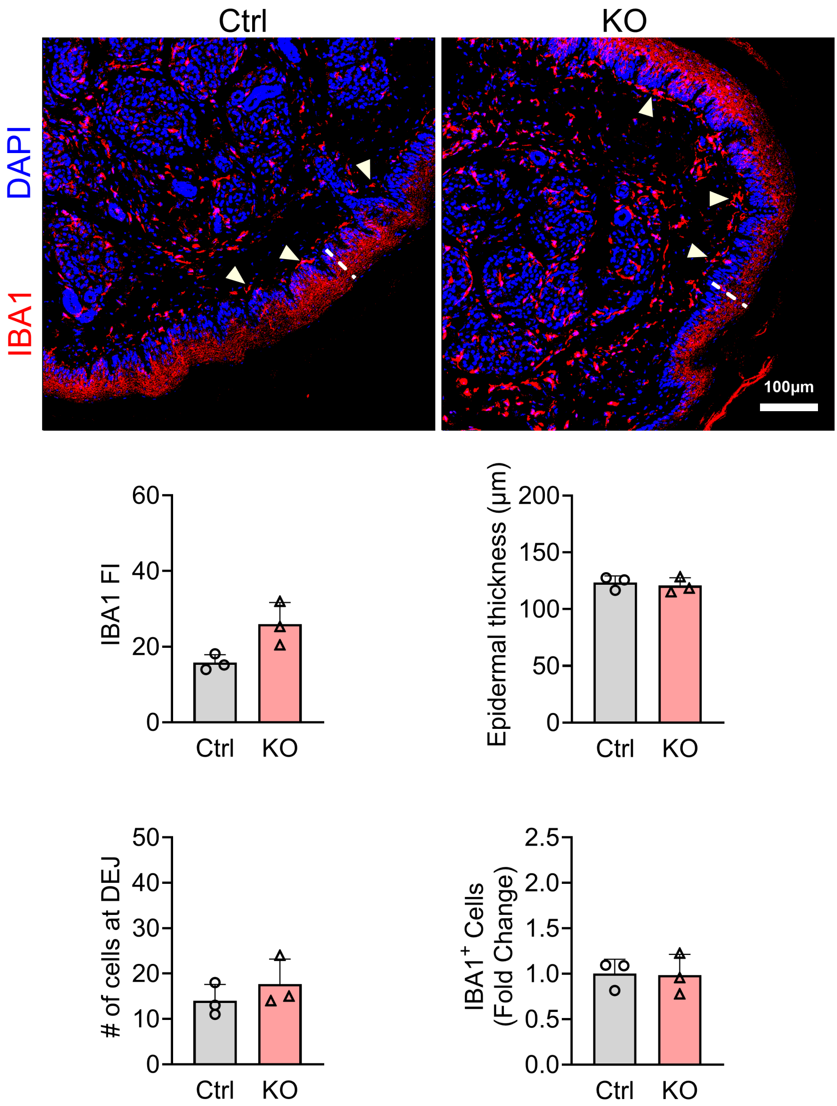

Supplementary Figure 4

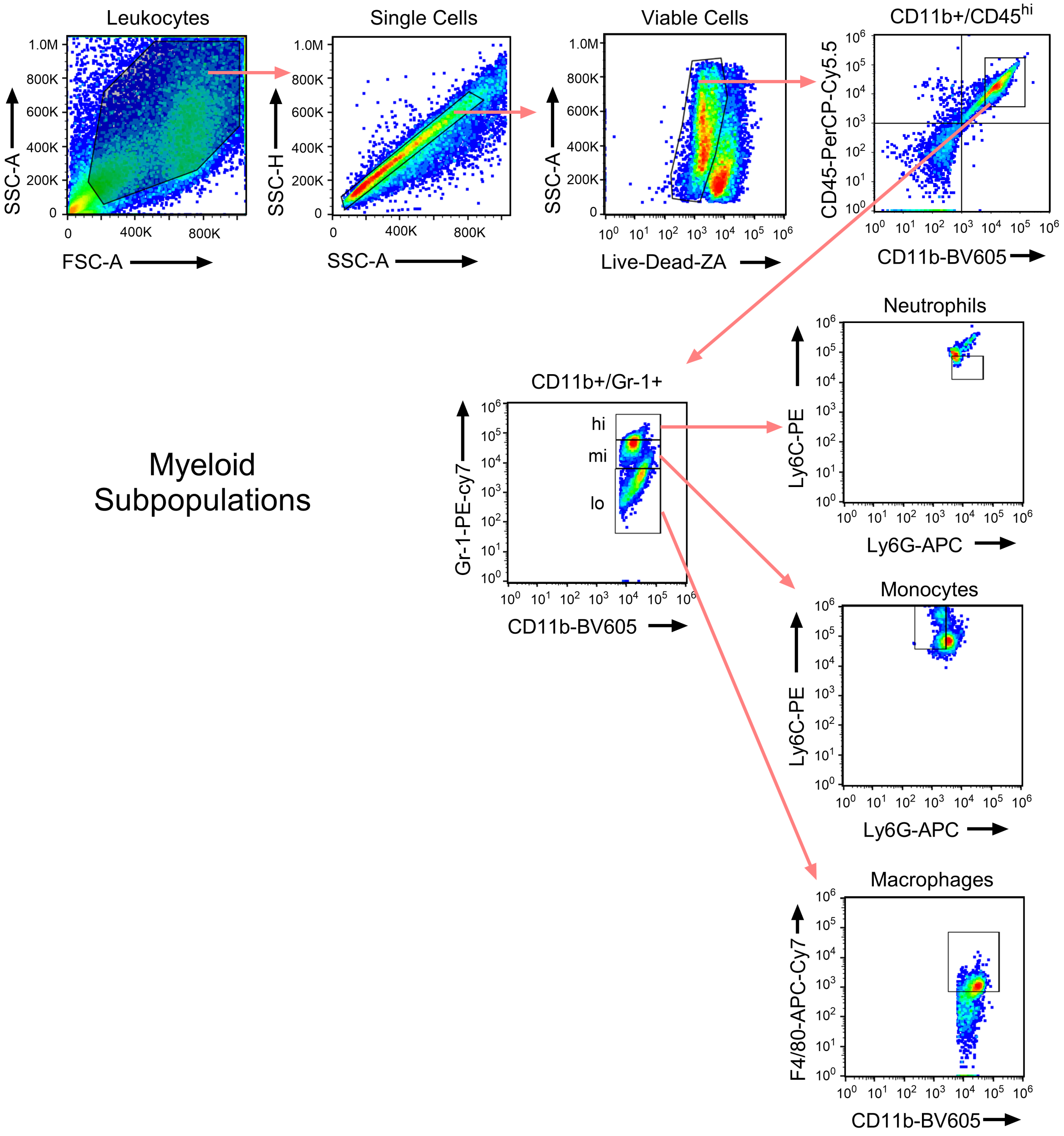

Supplementary Figure 5

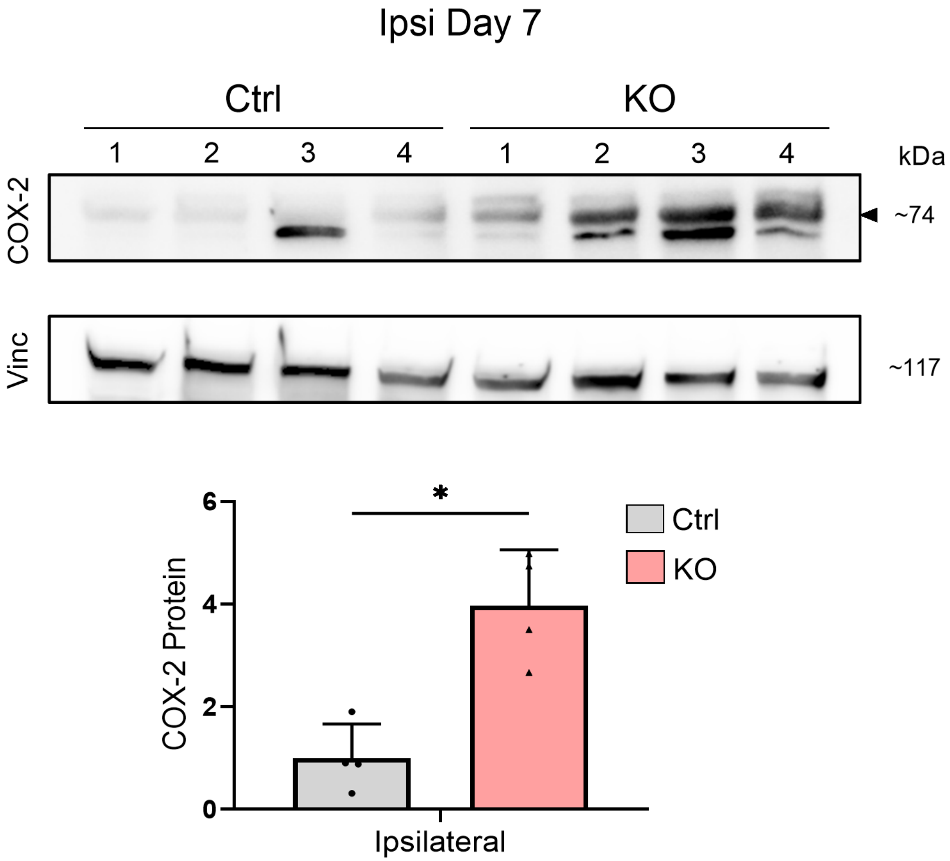

Supplementary Figure 6

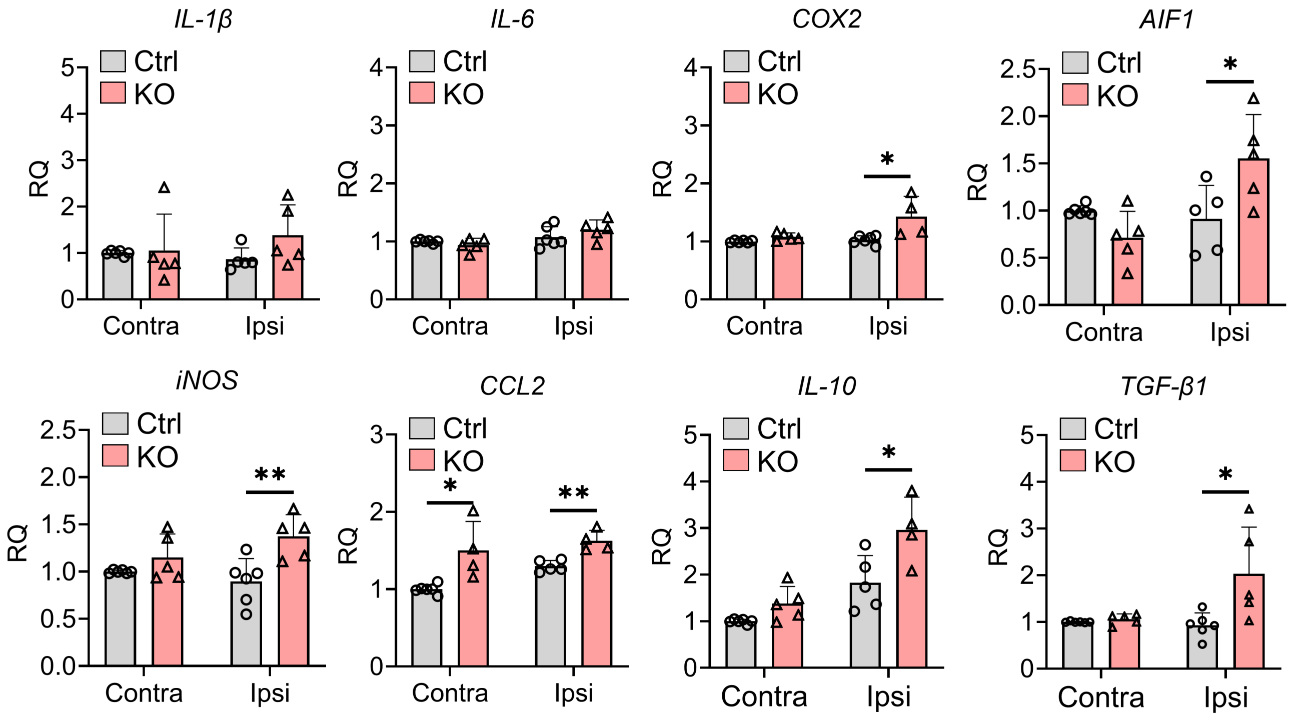

Supplementary Figure 7

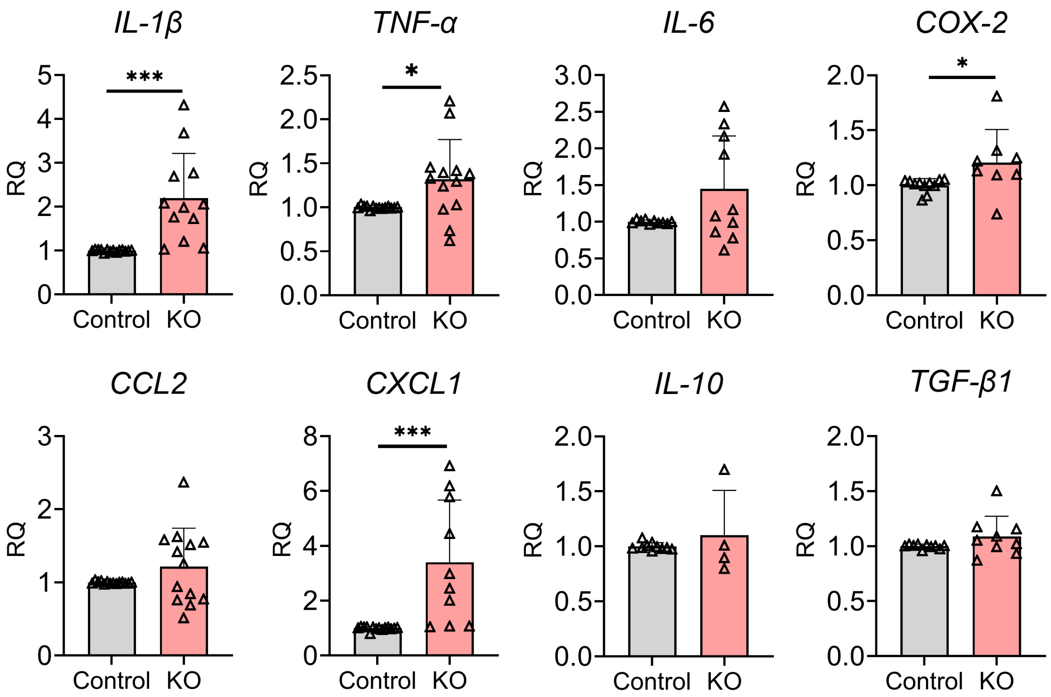

Supplementary Figure 8

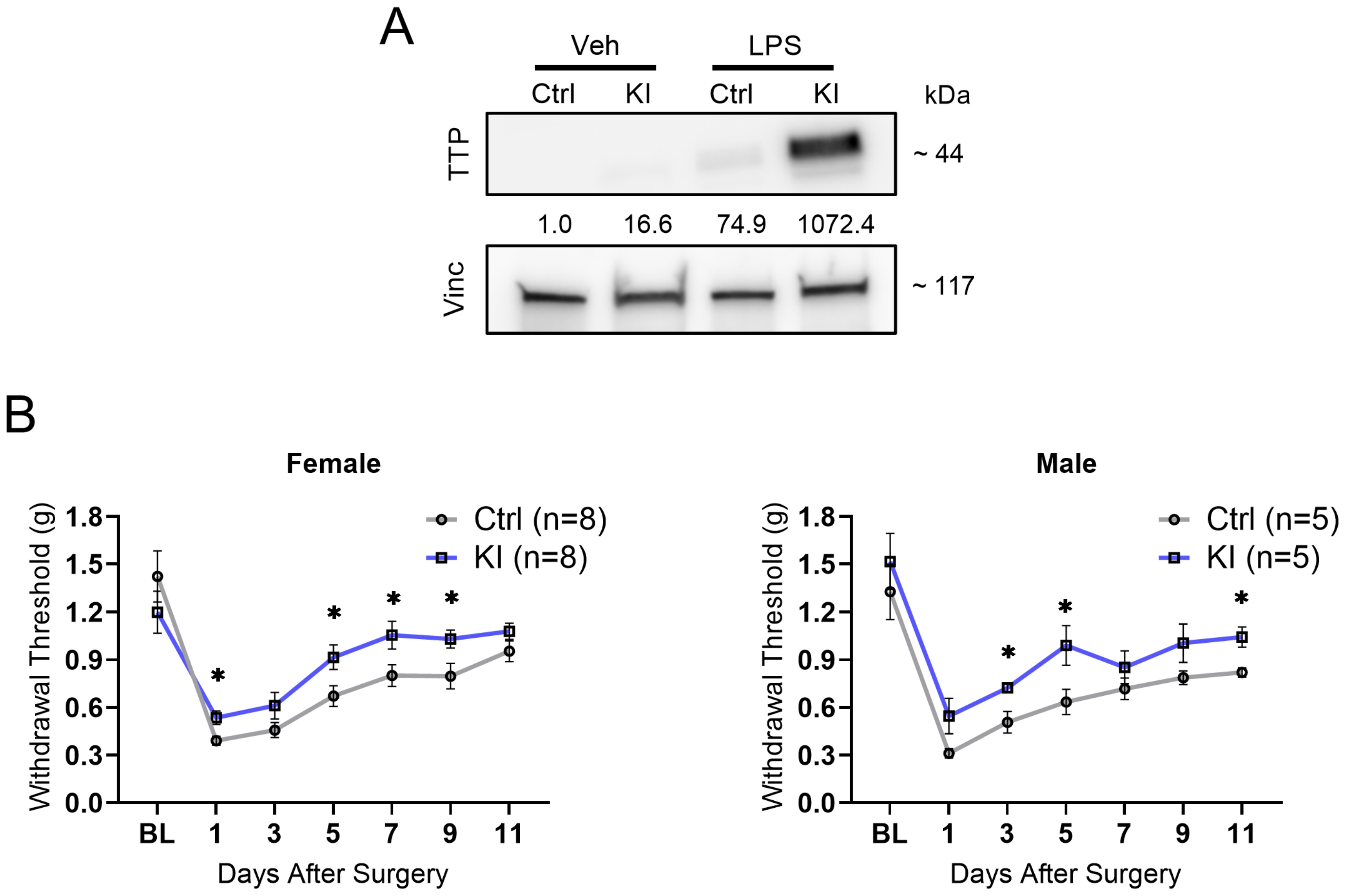

Supplementary Figure 9

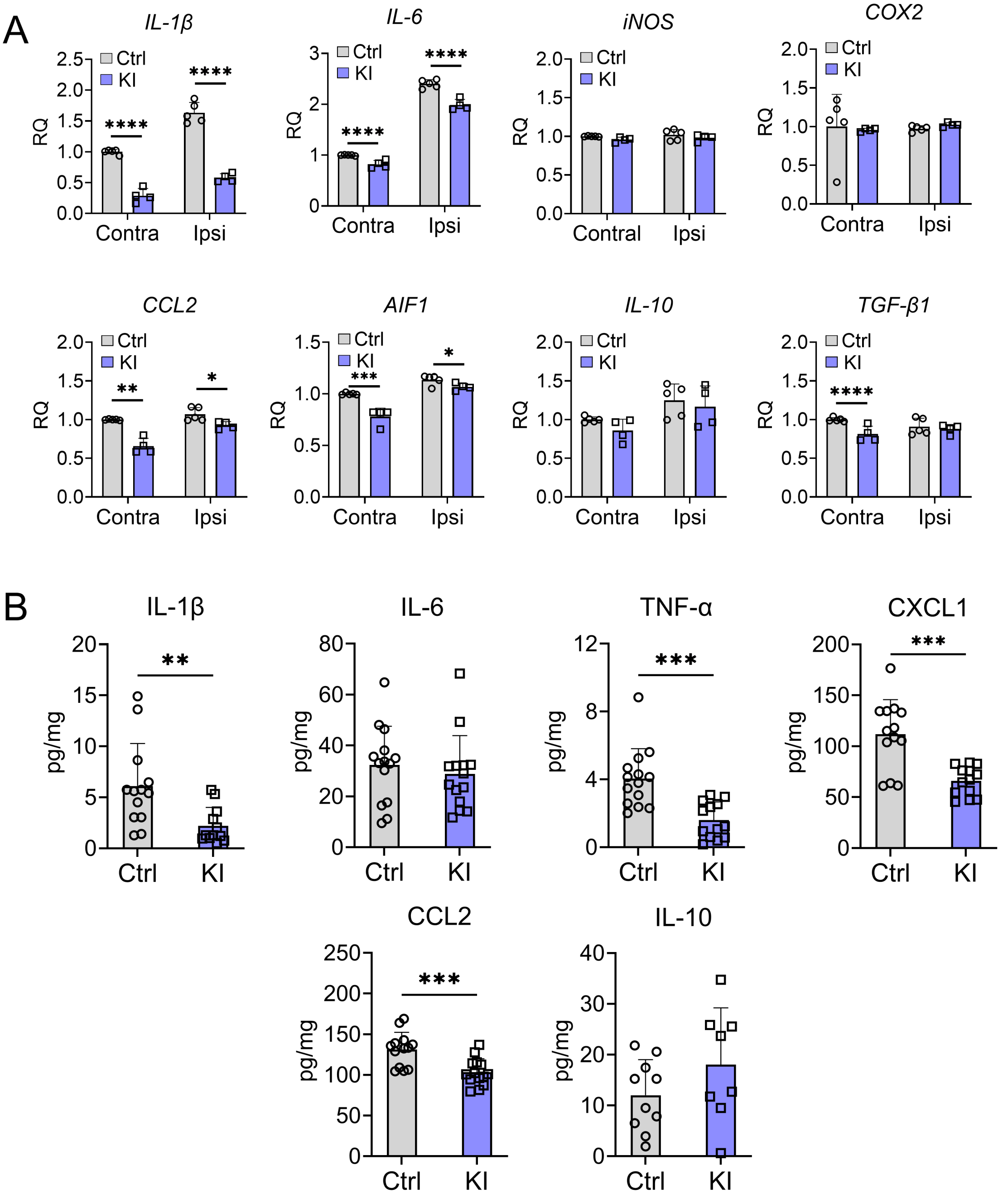

Supplementary Figure 10

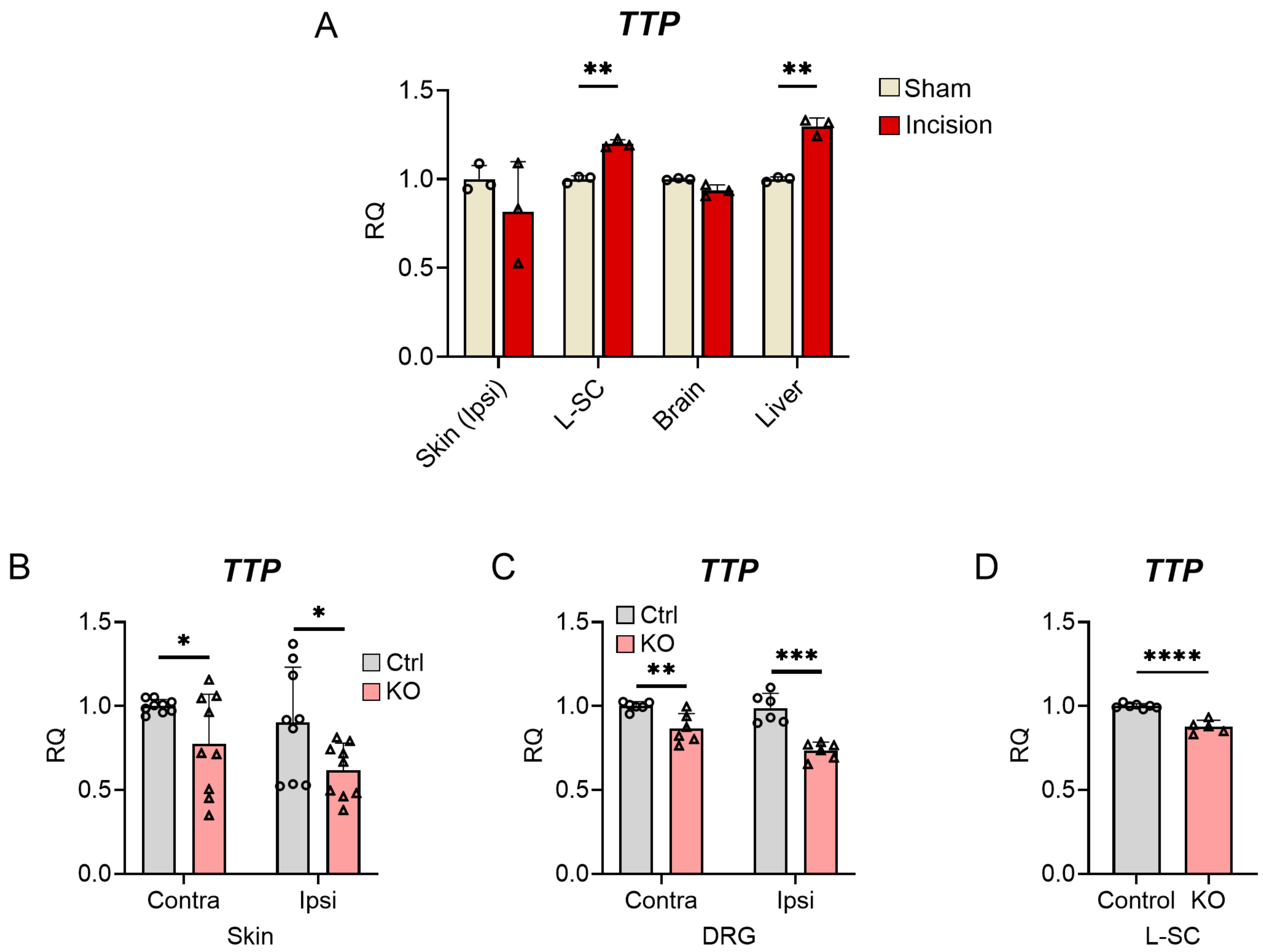

Supplementary Figure 11

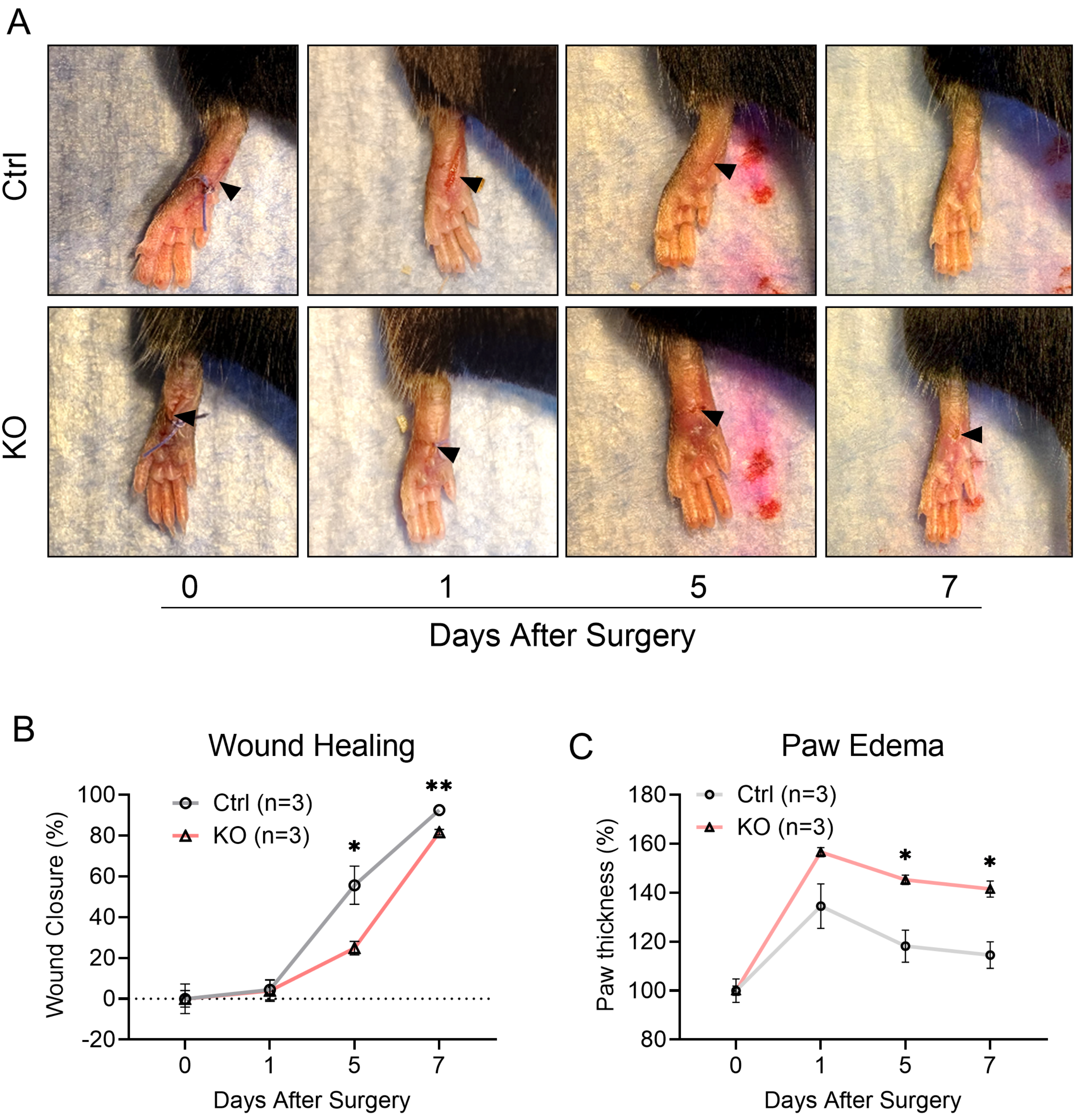

Supplementary Figure Legends

Supplementary Figure 1. Myeloid-specific TTP deletion exacerbates mechanical allodynia in both female and male mice following surgical incision or Complete Freund's adjuvant (CFA)-injection. (A) RNA levels of selected cytokines and chemokines were measured in BMDM, derived from TTP KO or floxed control mice treated with LPS. **P* < 0.05, unpaired two-tailed t-test, data represent three biological replicates (individual cellular experiment), each having two technical repeats. Error bars represent SD. **(B)** TTP KO and control female and male mice underwent plantar incision and were tested for mechanical allodynia (withdrawal thresholds) at 1, 3, 5, 7, 9 and 11 days. **P* < 0.05, ***P* < 0.01, Multiple comparison unpaired t-test with Welch's correction. Data represent mean ± SEM. **(C)** TTP KO and control mice were given CFA injection and tested for mechanical allodynia (withdrawal thresholds) at 1, 3, 5, and 11 days. **P* < 0.05, Multiple comparison unpaired t-test with Welch's correction. Data represent mean ± SEM.

Supplementary Figure 2. Surgical incision triggers epidermal thickening in TTP deleted mice. Ipsilateral skin tissue sections from a control and TTP KO mouse were stained using H&E staining kit to visualize skin pathology including epidermal thickening 24h post surgery. Scale bar, 100 μm.

Supplementary Figure 3. Sham-injured TTP KO mice show no difference in macrophage infiltration or epidermal thickness compared to littermate controls. (A) Ipsilateral skin tissue sections were immunostained with an anti-IBA1 antibody and counterstained with DAPI. White arrows indicate the $IBA1^+$ macrophages present at dermal-epidermal junction (DEJ). Scale bars,

100 μM. **(B)** Fluorescence Intensity (FI) of IBA1 immunoreactivity (left upper), epidermal thickness (right upper), number of IBA1 at DEJ (left lower) and total number of IBA1^{+} cells (right lower) were quantified in 3 controls and 3 TTP KO samples. Error bars represent SD.

Supplementary Figure 4. Gating strategy used to analyze myeloid subpopulations. Flow cytometry of incisional site was performed to identify myeloid cells in TTP KO mice. Gating strategy was used to determine macrophages, monocytes and neutrophils.

Supplementary Figure 5. TTP modulation of COX-2 at the incision site persists at 7 days. Western blotting of skin samples from the incision site was performed to detect COX-2 expression 7-days post incision in TTP KO mice floxed littermates were used as controls. Band intensity was quantified and adjusted for a loading control (vinculin). Data represent the mean ± SD. *$P < 0.05$, paired t-test.

Supplementary Figure 6. TTP KO mice have reduced expression of inflammatory mediators in DRG 7 days after incision. RNA levels of selected cytokines and chemokines were measured in DRG samples, both contralateral and ipsilateral to the incision site, 7 days post-incision. Each data point represents DRGs from 2-3 mice. *$P < 0.05$, **$P < 0.01$, unpaired two-tailed t-test, error bars represent SD.

Supplementary Figure 7. TTP KO mice have reduced expression of inflammatory mediators in L-SC 7 days after incision. RNA levels of selected cytokines and chemokines were measured by qRT-PCR in L-SC samples at 7 days post-incision. Each data point represents an individual mouse sample. *$P < 0.05$, ***$P < 0.001$; unpaired two-tailed t-test, error bars represent SD.

Supplementary Figure 8. TTP overexpression reduces mechanical allodynia following incision in both female and male mice. (A) Representative Western blot of BMDM from TTP KI or control mice treated with LPS. Antibodies used are shown to the left of the blot. Densitometry ratios of band intensities (TTP/Vinculin normalized to vehicle control) are shown below the TTP blot. **(B)** TTP KI and littermate controls underwent plantar incision and were tested for mechanical allodynia (withdrawal thresholds) at 1, 3, 5-, 7-, 9- and 11-days post-incision. *$P < 0.05$, Multiple comparison unpaired t-test with Welch's correction. Data represent mean ± SEM.

Supplementary Figure 9. TTP overexpression reduces the levels of inflammatory mediators in DRG and L-SC after surgical incision. (A) mRNA levels of selected cytokines and chemokines were measured in DRG of control and TTP KI samples, both contralateral and ipsilateral to the incision site, 1 day post incision. Each data point represents DRGs from 2-3 mice. *$P < 0.05$, **$P < 0.01$, ***$P < 0.001$, ****$P < 0.0001$; unpaired two-tailed t-test, error bars represent SD. **(B)** L-SC tissues from control and TTP KI mice were harvested 1 day post incision. Protein levels of selected cytokines and chemokines from these samples were analyzed by ELISA. **$P < 0.01$, ***$P < 0.001$; unpaired two-tailed t-test, error bars represent SD.

Supplementary Figure 10. Surgical incision leads to differential TTP expression in control and TTP KO mice. (A) TTP mRNA levels were assessed 1 day post-incision in ipsilateral skin, L-SC, brain, and liver of control mice. **(B-D)** TTP mRNA levels in skin (B), DRG (C), and L-SC (D) were compared between control and TTP KO mice 1 day post-incision. Each data point represents an individual mouse; $*P < 0.05$, $**P < 0.01$, $***P < 0.001$, $****P < 0.0001$; unpaired two-tailed t-test, error bars represent SD.

Supplementary Figure 11. Myeloid-specific TTP deletion impaired wound healing and enhanced paw edema post-incision in mice. (A) Representative paw photographs of TTP KO vs control mice post-surgical incision showing wound sites (arrows) and closure over time. **(B)** Wound healing progression (closure) in TTP KO vs control mice at days 0, 1, 5, 7 post-plantar incision**. (C)** Paw edema (thickness) in TTP KO vs control mice at same time points. $*P < 0.05$, $**P < 0.01$ (Multiple comparison unpaired t-test with Welch's correction). Data shown as mean ± SEM.
